# Important Poisonous Plants in Tibetan Ethnomedicine

**DOI:** 10.3390/toxins7010138

**Published:** 2015-01-14

**Authors:** Lijuan Ma, Ronghui Gu, Li Tang, Ze-E Chen, Rong Di, Chunlin Long

**Affiliations:** 1 College of Life and Environmental Sciences, Minzu University of China, Beijing 100081, China; E-Mails: maljsmile@126.com (L.M.); guronghui0812@163.com (R.G.); etangli@126.com (L.T.); chandice@126.com (Z.-EC.); 2 Department of Plant Biology and Pathology, School of Environmental and Biological Sciences, Rutgers University, New Brunswick, NJ 08901, USA; E-Mail: di@aesop.rutgers.edu; 3 Kunming Institute of Botany, Chinese Academy of Sciences, Kunming 650201, China

**Keywords:** poisonous plants, Tibetan ethnomedicine, toxins, aconitine, strychnine, scopolamine, anisodamine

## Abstract

Tibetan ethnomedicine is famous worldwide, both for its high effectiveness and unique cultural background. Many poisonous plants have been widely used to treat disorders in the Tibetan medicinal system. In the present review article, some representative poisonous plant species are introduced in terms of their significance in traditional Tibetan medicinal practices. They are *Aconitum* *pendulum*, *Strychnos nux-vomica*, *Datura* *stramonium* and *Anisodus tanguticus*, for which the toxic chemical constituents, bioactivities and pharmacological functions are reviewed herein. The most important toxins include aconitine, strychnine, scopolamine, and anisodamine. These toxic plants are still currently in use for pain-reduction and other purposes by Tibetan healers after processing.

## 1. Introduction

The Tibetan people have lived in Tibet (Xizang), Yunnan, Sichuan, Gansu, Qinghai and surrounding areas for many centuries. They have developed the Tibetan medical system based on the theory of “Four Tantras” (*rgyud bzhi* in the Tibetan language) and Tibetan medicine is still being used to treat various ailments in both urban and rural areas [1,2].

As one of the most famous and important ethnomedicines in China, the Tibetan medicine has a history of more than 2500 years and was originated during the pre-Buddhist era when the Tibetan region was ruled under the Kingdom of *Zang Xung* (*Xiang Xiong*, *Shang Shung* or *Zhang Zhung*). The Tibetan medicine, called *Sowa Rigpa* in the Tibetan language, is still effective and widely used, even though the western and traditional Chinese medicinal systems have been practiced for several decades in the Tibetan areas.

The earliest treatments included natural herbal remedies and ritual practices. When the shamans presented their superstitious worship rituals to treat disorders, they used herbal medicines in the forms of poultices and wraps, especially for wounds [3]. Living in the high elevation area with steep mountains, the Tibetan people would easily suffer from falls, fractures or other accidents. It became a priority to stop the pain when the shaman practiced their treatments. The pain-alleviation remedies normally contained toxins. Under the guidance of theories of traditional Tibetan medicine, the application of poisonous medicinal plants for some difficult diseases and severe acute diseases were evolved for thousands of years. The Tibetan healers still collect and prepare various poisonous plants for these remedies.

In Tibetan areas, poisonous plants are widely distributed from valleys to alpines, and from forests to meadows. Poisonous plants used in Tibetan medicine cover a large range of families, such as Polygonaceae (e.g., *Rheum*), Ranunculaceae (*Aconitum* and *Dalphinium*), Papaveraceae (*Meconopsis* and *Papaver*), Euphorbiaceae (*Euphorbia*), Thymelaeaceae (*Daphne*, *Stellera* and *Wikstroemia*), Fabaceae (*Oxytropis*), Loganiaceae (*Strychnos*), Asteraceae (*Liguraria* and *Saussurea*), Solanaceae (*Anisodus*, *Datura* and *Hyoscyamus*), Araceae (*Arisaema*), Liliaceae (*Veratrum*) and many others [4,5,6]. Most of these plants contain toxic constituents with biological activities.

## 2. Representatives of Poisonous Plants in Tibetan Medicine

**Figure 1:**
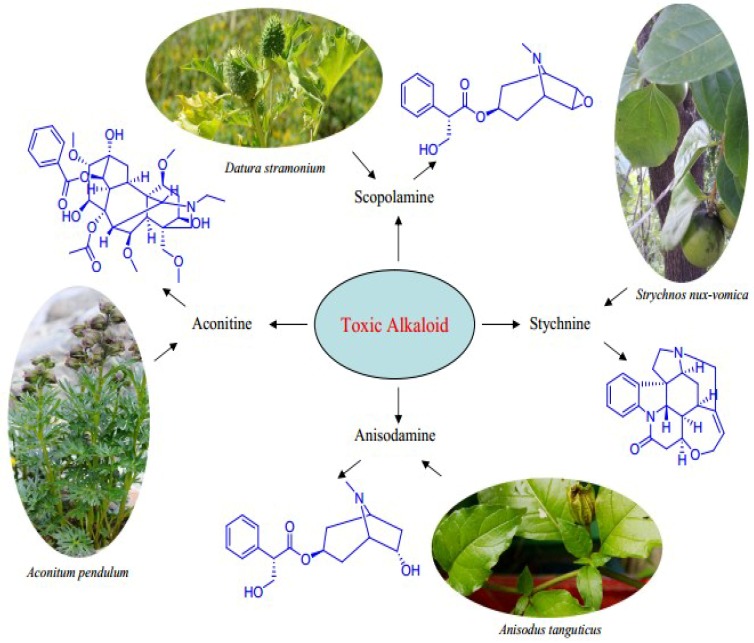
The most important toxic alkaloids from representatives of Tibetan poisonous medicine.

Based on the literature [4,5,6], we propose that the following four species are representatives of the commonly used poisonous plants in Tibetan medicine: *Aconitum* *pendulum* (Ranunculaceae), *Strychnos nux-vomica* (Loganiaceae), *Datura stramonium* (Solanaceae) and *Anisodus tanguticus* (Solanaceae) (Figure 1). Botanical and ethnopharmacological aspects of these four species are summarized in this review.

### 2.1. Aconitum pendulum

*Aconitum* is a large genus with more than 400 species, distributed in the temperate regions of the north hemisphere. There are 211 species in China, of which 166 are endemic to the country [7]. Most of the Chinese *Aconitum* species are found in Sichuan, Yunnan and Tibet [7,8,9].

In China, 76 *Aconitum* species have been used as herbal medicine and ethnomedicine. Most *Aconitum* species are very poisonous although the aconite roots of a few species have been consumed as root vegetables in China for a long time [10,11]. They are mainly used in treating plaque, sepsis, intoxication, cold- and immune suppression-induced ailments, rheumatoid arthritis, and various types of pain, such as migraine, swelling caused by trauma and fracture, and facial paralysis. Pharmacologically, they can be developed as analgesic, anti-rheumatic and anti-arrhythmic agents. The key obstacle for their extensive medical utilization may be attributed to their extremely high toxicity [12].

In Tibetan medicine, at least 15 species (taxa, including varieties) of *Aconitum* have been used for a long time [4,5,8,9,10]. They are divided into three ethnotaxa categorized by their color and toxicity of caudices, namely *Bang-Ga*, *Bang-Ma* (or *Bang-Se*), and *Bang-Na* in the Tibetan language (Table 1).

Among these 15 *Aconitum* species used in Tibetan medicine, *Aconitum* *pendulum* Busch is the most important and commonly used one, which was named *Manqin* in Tibetan or *Xueshang Yizhihao* in Chinese. The medicinal part is the dried caudex. *Manqin* is widely used in prescriptions for treating ankle pains, arthritis, traumatic injuries, influenza, blast epidemic, furuncle carbuncle and tumors [5]. *Manqin’s* botanical description is: Caudex obconical. Stem 26–100 cm. Proximal cauline leaves withered at anthesis, petiole 4–5 mm; leaf blade broadly ovate, 3.4–5.5 × 4.5–5.5 cm, both surfaces glabrous, 3–5-sect. Inflorescence 6–20 cm, 8–35-flowered; rachis and pedicels densely spreading yellow pubescent. Pedicels 2–6 mm, distally with 2 bracteoles. Sepals yellow, usually greenish, sometimes blue. Petals glabrous or sparsely pubescent; limb *ca*. 8 mm. Stamens glabrous or sparsely pubescent; filaments entire. Carpels 5, glabrous or ovary spreading pubescent. Seeds *ca*. 3 mm. Flowering from July to September. Growing in grassy slopes, forest margins; 2800–4500 m above sea level in southwest China [6].

**Table 1:** *Aconitum* species used in Tibetan medicine.

Category in Tibetan Medicinal System	Scientific Name	Distribution and Origin	Toxicity
*Bang-Ga* (with white caudices)	*Aconitum naviculare* (Brühl) Stapf	Tibet; Bhutan, NE India	Low
	*Aconitum tanguticum* (Maxim.) Stapf	SW & W China	Low
*Bang-Ma* or *Bang-Se* (with red or yellow caudices)	*Aconitum brunneum* Hand.-Mazz.	SW & W China	Low
	*Aconitum pulchellum* Hand.-Mazz.	SW China; Bhutan, NE India, Myanmar	Low
	*Aconitum pulchellum* var. *racemosum* W. T. Wang	Yunnan	Low
*Bang-Na* (with black caudices)	*Aconitum acutiusculum* var. *aureopilosum* W. T. Wang	Yunnan	High
	*Aconitum brachypodum* Diels	Sichuan, Yunnan	High
	*Aconitum* *bracteolosum* W. T. Wang	Yunnan	High
	*Aconitum dolichorhynchum* W. T. Wang	Yunnan	High
	*Aconitum flavum* Hand.-Mazz.	SW & W China	High
	*Aconitum forrestii* Stapf	SW China	High
	*Aconitum gezaense* W. T. Wang et L. Q. Li	Yunnan	High
	*Aconitum kongboense* Lauener	SW China	High
	*Aconitum pendulum* Busch	SW & W China	High
	*Aconitum sungpanense* Hand.-Mazz.	SW & W China	High

Processing is necessary in Tibetan ethnomedicine, especially for poisonous crude drugs. Before the caudices of *Aconitum* *pendulum* are used in medicine, they are soaked in water for 3–4 days, then cooked in boiling water for 4–6 h, followed by cutting into slices and drying. The processed caudices are still very poisonous, and can only be taken under the guidance of knowledgeable Tibetan healers.

### 2.2. Strychnos nux-vomica

The genus *Strychnos* L., with about 190 species, is distributed in the tropics and subtropics. Only 11 species are found in China. Most species in the genus are medicinal and poisonous [13]. Two *Strychnos* species, *S. nux-vomica* and *S. wallichiana* Steudel ex A. de Candolle (*S*. *pierriana* A. W. Hill), have been used in Tibetan medicine. *Strychnos nux-vomica* (called *Tumdgha* in Tibetan) is the most important medicinal plant in the genus although it is a domesticated species in China. Its seeds, as the *materia medica*, are mainly imported from India.

The botanical description of *Strychnos nux-vomica* L. is: Trees 25 m tall. Petiole 0.5–1.5 cm; leaf blade 5–18 × 4–12.5 cm, papery, basal veins 3–5. Thyrses axillary, 3-6 cm; peduncle puberulent; bracteoles pubescent. Flowers 5-merous. Pedicel puberulent. Calyx lobes ovate. Corolla greenish white to white, salverform, *ca*. 1.3 cm; tube longer than lobes; lobes narrowly ovate, *ca*. 3 mm. Stamens inserted at mouth or corolla tube; filaments very short; anthers elliptic, *ca.* 1.7 mm. Pistil 1–1.2 cm. Ovary ovoid. Style to 1.1 cm; stigma capitate. Berries orange when ripe, globose, 2–4 cm in diam., 1–4-seeded. Seeds orbicular to elliptic, 2–4 cm wide. Flowering in spring to summer. The seeds are used as medicine, which are very poisonous [13].

The *Strychnos nux-vomica* seeds must be processed before use in medicine. The major processing methods include cooking with tofu, stir-frying with soil or sands, boiling with vinegar, roasting, cauterizing with *Ephedra* or licorice (*Glycyrrhiza uralensis*), and others [14]. The seeds are commonly used to treat traumatic injuries, pains, anaesthesia, paralysis, and tumors by traditional Tibetan healers.

### 2.3. Datura stramonium

The genus *Datura* with 11 species originated from the Americas but is now widely distributed in the world. There are only 3 species in China, all of which are medicinal and poisonous [15].

The botanical description for *Datura stramonium* L. (commonly named *Mantuoluo*) is briefly summarized here: Herbs annual, 0.5–1.5 m tall. Stems often dark violet. Petiole 2–6 cm; leaf blade 5–20 × 4–l5 cm, veins 4–6 pairs. Flowers erect. Pedicel *ca.* 1 cm. Calyx tubular, 4–9 cm. Corolla white, yellowish, or pale purple, funnelform, 14–20 cm; limb 6–10 cm in diam. Anthers 1–1.2 cm. Capsules *ca*. 3 cm in diam. Seeds pale brown, *ca.* 3 mm in diam. Flowering and fruiting in March—December. It grows on grassy and sunny slopes 1200–2100 m above sea level, near houses, or cultivated for ornamental or medicinal purposes. *Datura stramonium* is very common in southern and southwestern China [15].

The whole plant, especially its seed, is toxic. The flowers are used as an anaesthetic. The seeds are used to treat ankle pains, asthma, cough, gastric convulsion, and traumatic injuries.

### 2.4. Anisodus tanguticus

There are only four species in the genus *Anisodus*, and all species are found in China [15]. Among these plants, *Anisodus tanguticus* (*Tangchong* in Tibetan language) is the most important species in Tibetan medicine.

The botanical characteristics of *Anisodus tanguticus* (Maxim.) Pascher is as follows: Herbs perennial, 40–100 cm tall. Roots stout. Petiole 1–3.5 cm; leaf blade 8–20 × 2.5–9 cm. Flowers nodding or erect; pedicel 1.5–8 cm. Calyx 2.5–4 cm. Corolla purple or dark-purple, sometimes pale yellow-green, resembling calyx in shape, 2.5–3.8 cm. Stamens less than half as long as corolla; filaments *ca*. 0.8 mm; anthers oblong, 5–6 mm. Style 1.2 cm. Fruiting pedicel 6–8 cm, erect. Fruiting calyx *ca*. 6–7.5 cm. Capsule *ca*. 2 cm in diam. Flowering in May–June, and fruiting in July–August. It grows on sunny grassy slopes; 2000–4400 m, mainly distributed in Gansu, Qinghai, NW and SW Sichuan, E Tibet, NW Yunnan, and Nepal [15].

The local Tibetan healers use the roots of *Anisodus tanguticus* to treat pains, ulcers, colitis, gallstone, traumatic injuries, catagma and hemorrhage. In autumn or winter, they collect roots and cut them into slices for drying. Because of high toxicity, only a small amount of *Anisodus tanguticus* roots can be used externally.

## 3. Chemical Constituents of Important Poisonous Plants in Tibetan Medicine

In recent years, the chemical constituents of these representative poisonous plants in Tibetan medicine have been studied. In *Aconitum pendulum*, alkaloids, steroids and glycosides have been isolated and determined [16,17,18,19,20]. Alkaloids, iridoids and fatty acids were isolated from *Strychnos nux-vomica* [21,22,23]. *Datura stramonium* is rich in alkaloids, flavanoids, withanolides as well as sesquiterpenes [24,25,26,27]. The alkaloids have also been extracted from *Anisodus tanguticus* [28]. Although numerous compounds have been isolated from these plants, alkaloids and their derivatives are generally considered as the key toxic and medicinal constituents.

### 3.1. Alkaloids from Aconitum pendulum

**Figure 2:**
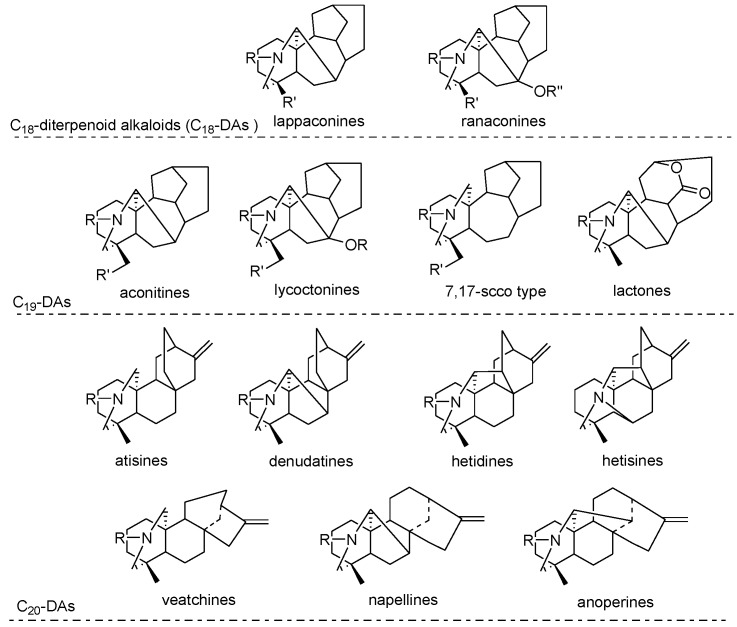
Main skeletal types of diterpenoid alkaloids in *Aconitum*.

*Aconitum* species are rich in diterpenoid alkaloids, a group of highly toxic and medicinal constituents, which have been widely studied for their complex structures, thought-provoking chemistry, and noteworthy bioactivities [29]. Based on the structure of diterpenoid alkaloids (DAs) isolated from *Aconitum*, they are commonly divided into three skeletal types: C18, C19 and C20 alkaloids [12,30] (Figure 2), of which C18-diterpenoid alkaloids are the major compounds.

**Figure 3:**
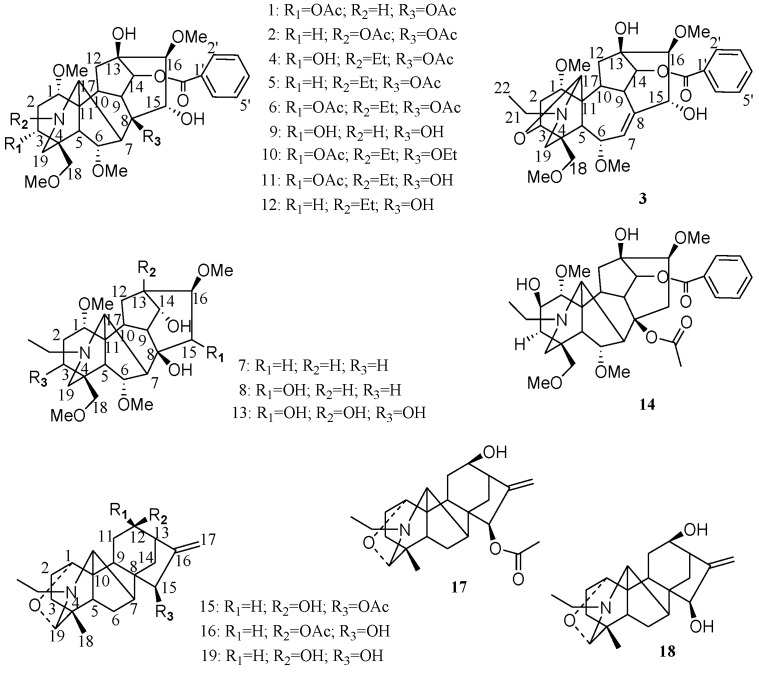
Chemical structures of alkaloids isolated from *Aconitum pendulum*.

Recently, some DAs have been isolated from *A. pendulum*. Wang *et al.*, obtained three new C19-nor-diterpenoid alkaloids from the roots of *A. pendulum*, and named them *N*-deethyl-3-acetylaconitine (**1**), *N*-deethyldeoxyaconitine (**2**) and secoaconitine (**3**) [17]. In addition, they isolated aconitine (**4**), deoxyaconitine (**5**), 3-acetylaconitine (**6**), neoline (**7**), 15-α-OH-neoline (**8**), benzoylconitine (**9**), polyschistine A (**10**), polyschistine D (**11**), benzoyldeoxyaconitine (**12**) and aconine (**13**) from 95% EtOH extract of its roots [18] (Figure 3). Actually, as early as 1997, 10 alkaloids were isolated from the roots of *A. pendulum*, including 2-hydroxydeoxyaconitine (**14**), 12-epiacetyldehydrolucidusculline (**15**), 12-epiacetyldehydronapelline (**16**), dehydrolucidusculline (**17**) and dehydronapelline (**18**). 12-epidehydronapelline (**19**) and compounds **10** and **14** were new alkaloids [19] (Figure 3).

**Figure 4:**
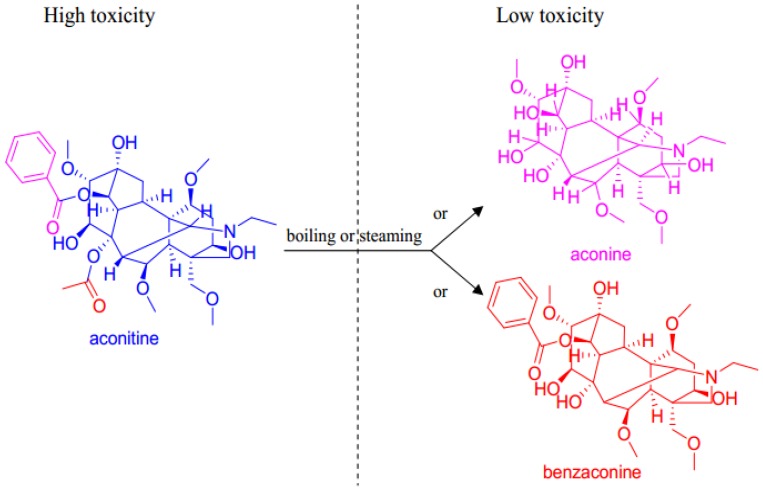
Change of aconitine during heat-processing.

In these alkaloids isolated from *A.* *pendulum*, aconitine has attracted the most attention for its high toxicity and wide range of bioactivities. It is a diester diterpenoid alkaloid, sharing the common C19-norditerpenoid skeleton. Structure-activity relationship research indicated that its high toxicity is attributed to the acetyl group at C8, the hydroxyl group at C13, 4 methoxyl groups at C1, C6, C16 and C18, and the benzoylester group at C14 [31]. It needs heat-processing with boiling or steaming for the raw *A. pendulum* caudices before they are used in Tibetan medicine prescriptions. During the heat-processing, some of the aconitine alkaloids of diester-type are converted into benzoylaconines or aconines, which have lower toxicity compared to aconitine [31,32] (Figure 4).

### 3.2. Alkaloids in Strychnos nux-vomica

The seeds of *Strychnos nux-vomica* are called *Maqianzi* in Chinese folk medicine, and *Tumdgha* in Tibetan medicine. They are commonly used for anaesthesia and treating traumatic injuries, pains, paralysis, and tumors by traditional Tibetan healers. In 1971, it was shown that alkaloids were the major bioactive components of this plant [33]. Since then, more and more alkaloids have been found in this species (Table 2). These alkaloids are toxic, and strychnine is the most abundant and has the highest toxicity. Makarovsky *et al.* pointed out that only 30 to 120 mg strychnine could kill a person [34]. Due to the high toxicity, the seeds of *Strychnos nux-vomica* must be processed before clinical practice. During processing, the toxic alkaloids are converted into their isoforms or nitrogen oxidation derivatives, which are more or equally as potent and less toxic than their parent alkaloids [35,36].

**Table 2:** Main alkaloids from *Strychnos nux-vomica*.

No.	Alkaloids	Tissue	Reference
1	Strychnine	Seeds, fruits	[37,38,39]
2	Brucine	Seeds	[37,38]
3	β-colubrine	Seeds	[37,38]
4	Icajine	Seeds	[37,38]
5	16-Hydroxy-α-colubrine	Seeds	[37]
6	Brucine-*N*-oxide	Seeds	[37,38]
7	Strychnine-*N*-oxide	Seeds	[37,38]
8	Vomicine	Seeds, fruits	[37,38,39]
9	Novacine	Seeds	[37,38]
10	Pseudostrychnine	Seeds	[37,38]
11	Pseudobrucine	Seeds	[37]
12	Isostrychnine	Seeds	[37,38]
13	Isobrucine	Processed seeds, seeds	[37,38]
14	Isobrucine-*N*-oxide	Processed seeds, seeds	[37,38]
15	Isostrychnine-*N*-oxide	Processed seeds, seeds	[37,38]
16	2-Hydroxy-3-methoxystrychnine	Processed seeds	[37]
17	4-*N*-hydroxymethyl-strychnidin-17-acetic acid	Seeds	[40]
18	10,11-Dimethoxy-4-*N*-hydroxymethyl strychnidin-17-acetic acid	Seeds	[40]

**Figure 5:**
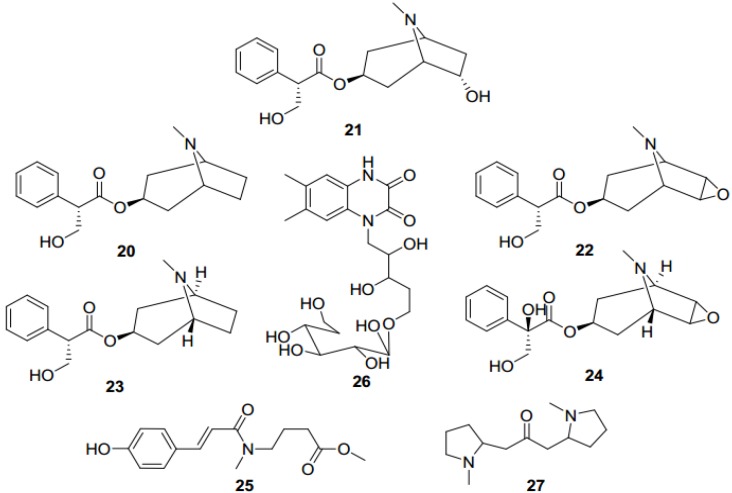
Main alkaloids from *Datura stramonium* and *Anisodus tanguticus*.

### 3.3. Alkaloids from Datura stramonium and Anisodus tanguticus

*Datura stramonium* and *Anisodus tanguticus* are important medicinal plants in the Solanaceae family in Tibetan medicine. Chemically, these two species are similar due to the abundant tropane alkaloids, which are characteristic in the Solanaceae family [41].

Many tropane alkaloids have been found in *D. stramonium*, including hyoscyamine (**20**), anisodamine (**21**), scopolamine (**22**), atropine (**23**) and anisodine (**24**) [24,42]. Recently, two novel amide alkaloids were separated from the alkaloidal fraction of *D. stramonium*. They are (*E*)-methyl 4-(3-(4-hydroxyphenyl)-*N*-methylacrylamido) butanoate (**25**) and 6,7-dimethyl-1-d-ribityl-quinoxaline-2,3(1H,4H)-dione-5'-*O*-β-d-glucopyranoside (**26**). This was the first time that amide alkaloids were reported in this species [25]. In *A. tanguticus*, hyoscyamine, anisodamine, scopolamine, tropine and cuscohygrine (**27**) were also isolated (Figure 5) [28,43].

Although the tropane alkaloids of these two plants are similar, their characteristic alkaloids are different. The main toxic topane alkaloids are scopolamine in *D. stramonium*, and anisodamine in *A. tanguticus*. These two alkaloids are highly toxic and greatly diversified in their pharmacological activities.

## 4. Biological Activities of Chemicals from Important Poisonous Plants in Tibetan Medicine

Owing to the strong toxicity and crucial traditional medicinal value, various therapeutic effects of the representatives of poisonous plants and their compounds have been investigated. Most commonly reported biological activities are antinociceptive, improving cardiovascular, anti-inflammatory, anticholinergic, antispasmodic and anticancer properties. In this review, the main biological activities of aconitine, strychnine, scopolamine, and anisodamine are summarized.

### 4.1. Biological Activities of Aconitine

Aconitine, a C19 diterpenoid alkaloid, has been found to possess significant antinociceptive, cardiovascular beneficial and anti-epileptiform effects [31,44,45]. Interestingly, these activities are mainly attributed to the effect of aconitine on voltage-gated Na^+^ channels.

In 1980, Catterall elucidated the antinociceptive mechanism for aconitine. Briefly, aconitine binds to site 2 of Na^+^ channels with high affinity, causing a sustaining activation of Na^+^ channels. Due to persistant Na^+^ channel activation, cells are depolarized by a permanent Na^+^ influx leading to inexcitability [46]. Structure-activity relationship analysis showed that aconitine contributed the benzoyl ester side chain in C-14 to activate the Na^+^ channel [47].

The initial research on aconitine toxicity focused on the cardiovascular-beneficial effect (arrhythmogenic), which is one of the factors contributing to fatal intoxications after ingesting *Aconitum*. Aconitine, binding with high affinity to the open state of Na^+^ channels at site-2, was found to delay the final repolarization of action potential of cardiac cells, leading to premature or triggered excitations [45]. The final inexcitability might lead to heart arrest. The substituents including β-acetate on C-8, β-OH on C-13, α-aroyl on C-14, were considered as the key arrhythmogenic factors [45].

Ameri’s group has contributed much research on the inhibition of neuronal activity and anti-epileptiform activity of some diterpene alkaloids in rat hippocampal slices. Their studies showed that aconitine (1 μM) completely suppressed both epileptiform activity and normal neuronal activity, whereas lappaconitine (10 μM) and 6-benzoylheteratisine (10 μM) showed anti-epileptiform activity by sparing normal neuronal activity [44]. Furthermore, the anti-epileptiform activity of these alkaloids was in line with the blockade of Na^+^ channels since Na^+^ channels were associated with the origin of the abnormal activity in epilepsy. In addition, anti-epileptic studies indicated that an aromatic substituent in these diterpene alkaloids is essential for their anti-epileptic activity [45].

Dosage needs to be taken into consideration particularly in medicinal practice due to aconitine’s potential toxicity. It has been shown that at the concentration of 30 nM, aconitine showed strong arrhythmogenic activity, an antinociceptive effect (ED_50_: 0.06 mg/kg) and high acute toxicity (LD_50_: 0.15 mg/kg) [47]. The estimated lethal dose was 2 mg of aconitine, 1 g of the raw aconite plant or 5 mL of aconite tincture (an medicinal wine made by local people which can be absorbed via the skin into systemic circulation) [48,49].

### 4.2. Biological Activities of Strychnine

Strychnine is an important indole alkaloid with a strychnan group, which has shown various biological activities.

Studies have been focused on the anti-tumor activities of strychnine. A few human cancer cell lines have been used to study the cytotoxic or anti-proliferative effects of strychnine. These cell lines, including SMMC-7721, HepG2 and RPMI-8226, exhibited growth inhibition at different levels when incubated with strychnine [50,51,52]. In 2009, the *S. nux-vomica* root extract was shown to exhibit anti-proliferative and cytotoxic activity in RPMI-8226 cells, which was attributed to strychnine and brucine. The morphological assessment showed significant apoptosis of the cells. Cell cycle analysis revealed that these cells stayed at the sub-G0/G1 phase. Treated RPMI-8226 cells were also shown to have disrupted mitochondrial membrane potential and leaky mitochondrial cytochrome C [52].

Angiogenesis is an important target of numerous chemo-defensive molecules, which control the angiogenic switch in pre-malignant tumors. Angiogenesis usually co-exists with inflammation in a variety of pathological states. The anti-tumor mechanism of strychnine is not completely known. In 2013, Saraswati and Agarwal studied the relationship between stychnine and inflammatory angiogensis in mice. They found that strychnine significantly reduced the main components of vascularization (haemoglobin content), macrophage recruitment (*N*-acetylglucosaminidase activity), and the levels of vascular endothelial growth factor (VEGF), tumor necrosis factor (TNF)-α and transforming growth factor (TGF-β). Their results indicated that strychnine could inhibit inflammatory angiogenesis via downregulation of VEGF, TNF-α and TGF-β in mice [53]. Recently, the effects of strychnine on zebrafish embryos was studied, and it was shown that strychnine at 200 μmol/L induced apoptosis, and the ratio of Bax/Bcl-2 and p53 mRNA expression was significantly altered [54]. These studies provided the basis for further exploring the potential pharmacological actions of strychnine.

Strychnine is also an analgesic and belongs to the group of analeptics. At low doses, strychnine can activate certain central nervous cells and stimulate vasomotor and respiratory centers. It can also induce convulsions by tetanizing activity [55].

The effects of strychnine on the nervous system are mainly due to its extremely potent glycine antagonist property. It was reported that strychnine not only blocked postsynaptic receptors of the inhibitory neurotransmitter glycine, but also played a role in presynaptic action restraining release of the inhibitory neurotransmitter in the spinal cord and motoneurons. This action of strychnine results in unrestrained excitatory synapses, leading to a series of abnormal actions, including motor disturbance, increased muscle tone, hyperactivity of sensory, visual and acoustic perception. At high doses, strychnine will result in tonic convulsions and death directly through spinal paralysis or respiratory or cardiac arrest [55].

Although strychnine is regarded as a highly toxic compound, a few significant bioactivities have been demonstrated. Its safety dose is 1–3 mg, which can increase spinal reflex with no effect on the respiratory and circulation systems. When the dose of strychnine is increased to 5–10 mg, the spinal reflex is greatly increased and muscles are tautened. More than 10 mg of strychnine may result in dyspnoea, anxiety, tonic convulsions and even death.

### 4.3. Biological Activities of Scopolamine

Scopolamine, a tropane alkaloid, is the active ingredient responsible for analgesia, amnesia and motion sickness [56]. It has an antagonistic action on muscarinic acetylcholine receptors in both peripheral and central nervous systems (CNS), exhibiting anticholinergic and spasmolytic properties [57].

Traditionally, scopolamine was commonly used in the study of neuropsychopharmacology as a standard or reference compound for inducing dementia- and age-related cognitive deficits in healthy humans and animals [58,59]. In 1974, it was first proposed that scopolamine simulated a few cognitive dysfunctions, like aging and dementia performance when administered to healthy volunteers [60]. Subsequently, numerous articles reported on the cognitive impairment induced by scopolamine (Table 3). To date, scopolamine has been employed as a common standard drug to estimate the activities of new substances such as imperatorin [61], biperiden [62], pioglitazone [63], and to test models and methods designed to study cognitive dysfunction (e.g., Alzheimer’s disease), in functional magnetic resonance imaging (fMRI) [64], and acupuncture [65].

As mentioned above, the bioactivity or toxicity mechanism of scopolamine may be attributed to its potent competitive muscarinic antagonist characteristics. Scopolamine can induce malfunction in the CNS’s memory circuits and the regulation of the cholinergic neuronal pathway. Moreover, it down-regulates the expression of cAMP-response element-binding protein (CREB) in the brain and brain-derived neurotrophic factor (BDNF) [65].

**Table 3:** Effects of scopolamine for cognitive impairment (Klinkenberg & Blokland, 2010 [59]).

Cognitive Impairment (CI)	Specific Behavior	Pharmacological Activities Phenomenon	Reference
Non-behavioral CI	Pupil diameter, salivation and smooth muscle function	Dose-dependent increase in pupil size	[66]
		Reduces salivation	[67]
		Induced gastrointestinal distress	[59]
	Electroencephalogram	Decreased low voltage fast activity	[68]
		Induced disturbances in gamma oscillations	[69]
Behavioral CI	Locomotor activity and motor learning	Increased locomotor activity	[70,71]
	Anxiety	lowered the number of transitions to the light side	[72]
	Stimulus discrimination	Implicated the (dorsal) hippocampus and cortex	[73,74]
	Attention	Impaired the maintenance of attention	[75]
	Learning and memory	Interfere with short-term memory	[76]

### 4.4. Biological Activities of Anisodamine

Anisodamine is isolated from *Anisodus tanguticus*, an important poisonous plant in Tibetan medicine. As a peripheral muscarinic antagonist, numerous pharmacologic activities, such as cognition improvement [77,78], anti-inflammation [79] and relieving spasms [80,81], have been demonstrated.

As a natural muscarinic acetylcholine receptor (mAChR) antagonists, anisodamine has lower toxicity and weaker effect on the central nervous system than scopolamine, although both of them belong to tropane alkaloids and are considered as mAChR antagonists [77]. Anisodamine has been reported to improve learning and memory in the avoidance response of rats after medial frontal cortex damage or acute cerebral ischemia and reperfusion [82]. Wang *et al.* found that normal mice did not suffer from memory deficits when anisodamine was administered 11 times at 20 mg/kg [78]. Recently, anisodamine administration resulted in no observable cognitive deficit, in contrast to scopolamine, and instead improved cognition, at a 40-fold higher dose than scopolamine [77].

There is only one difference in the structures of anisodamine and scopolamine. At C6, anisodamine has an OH group, while there is an oxygen bridge between C6–C7 in scopolamine. Therefore, it is possible that the lipophilic solubility of anisodamine is decreased by the OH group at C6, reducing its permeability, which may lead to less cognitive impairment. Neuropsychopharmacological research demonstrated that anisodamine did not influence the formation of long-term potentiation (LTP) in the CA_1_ region of rat hippocampus but scopolamine did. In addition, the binding affinity of anisodamine to mice brain mAChR was much lower than that of scopolamine [77]. These findings indicated that the poor blood brain barrier permeability of anisodamine contributed to its lower effectiveness on cognition impairment and LTP depression.

It was reported that anisodamine has the clinical applications to treat infectious shock, rheumatoid arthritis, glomerulonephritis, gastrointestinal colic and hemorrhagic necrotic enteritis [78], indicating its strong impacts on inflammation. In 2009, Sprague-Dawley rats injected with lipopolysaccharide (LPS), α7nAChR-deficient mice and RAW264.7 cells were used to study the anti-shock effect of anisodamine. The results showed that the anti-shock effect was attributed to the inhibitory effect on tumor necrosis factor-α (TNF-α) and interleukin-1β (IL-1β) [83]. Additionally, research from Zhou and colleagues demonstrated the anti-inflammation of anisodamine in collagen-induced arthritis in mice [84].

Anisodamine has been widely used to relieve microvascular, intestinal and airway smooth muscle spasms. Recent reports showed that the proliferation and tracheal contractility of smoke extract-induced airway smooth muscle cell could be suppressed and reversed by anisodamine, revealing its effect on relieving airway spasms [80]. Another investigation also demonstrated its similar effect on cardiac myocytes. Norby *et al.* declared that anisodamine could inhibit cardiac contractions and also inferred that the mechanism may be attributed to NO (nitric oxide) production and cholinoceptor antagonism [81].

## 5. Conclusions

Tibetan ethnomedicine has an important status both in traditional Chinese medicine and in the world medicine system. Poisonous plants, widely distributed in the Tibetan Plateau and neighboring areas, are significant sources of Tibetan medicine. In this review, four representative poisonous plants, *Aconitum* *pendulum*, *Strychnos nux-vomica*, *Datura stramonium* and *Anisodus tanguticus* are described in detail, including morphology of the plants, and the ethnopharmacology, toxicity, chemical constituents and bioactivity of the toxic compounds from these plants.

Although the representative plants possess various medicinal values, their high toxicity must be carefully evaluated. New methods and techniques should be adopted to improve their safety and availability. The process technologies and chemical conversions should be further studied. Although many compounds have been isolated from these plants, many other constituents need to be discovered. As for aconitine, strychnine, scopolamine and anisodamine, many of their significant bioactivities have been demonstrated. However, most of their modes of actions are still unknown. More studies are necessary to characterize their specific functions. In addition, synthetic strategies need to be considered to obtain specific compounds with higher bioactivities than their natural counterparts.
